# Geriatric Telehealth: A Standardized Patient Case for Medical Students

**DOI:** 10.15766/mep_2374-8265.11345

**Published:** 2023-09-12

**Authors:** Lindsay A. Wilson, Brianna Harder, Casey Kelley, Ross Powell, Megan Foster, Ellen Roberts

**Affiliations:** 1 Associate Professor, Division of Geriatrics, Department of Medicine, University of North Carolina at Chapel Hill School of Medicine; 2 Assistant Professor, Division of Geriatrics, Department of Medicine, University of North Carolina at Chapel Hill School of Medicine; 3 Research Assistant, Division of Geriatrics, Department of Medicine, University of North Carolina at Chapel Hill School of Medicine; 4 Resident Physician, Department of Internal Medicine, East Carolina University; 5 Resident Physician, Department of Family Medicine, University of Virginia School of Medicine

**Keywords:** Case-Based Learning, Geriatrics, Standardized Patient, Telehealth

## Abstract

**Introduction:**

The COVID-19 pandemic has necessitated the rapid expansion of telemedicine. However, there has been minimal coverage of telemedicine in traditional undergraduate medicine curricula. Telemedicine presents specific challenges in the geriatric population, including unfamiliarity with technology, cognitive and sensory barriers, inclusion of family and/or caregivers, multimorbidity, and a high degree of medical complexity.

**Methods:**

We developed a workshop to allow rising third-year medical students to practice a telemedicine patient encounter while developing skills for assessing and communicating with geriatric patients. This 90-minute workshop consisted of an introductory didactic presentation and a standardized patient activity for small groups of two to five students. Students’ level of comfort with telemedicine for assessment of geriatric patients was evaluated with a pre- and postsurvey.

**Results:**

Fifty-eight students participated in the workshop and completed the surveys (presurvey = 58, postsurvey = 40), with roughly half (52%) reporting prior experience with telemedicine. A 5-point Likert-type scale (1 = *very uncomfortable,* 5 = *very comfortable*) was used. Students reported statistically significant increases in comfort using telemedicine (presurvey = 3.1, postsurvey = 3.9, *p* < .001) and using telemedicine for patients ≥65 years (presurvey = 2.8, postsurvey = 3.9, *p* < .001) after completing the workshop.

**Discussion:**

Medical students’ comfort levels using telemedicine and caring for patients ages 65 and older with a telehealth visit improved after participating in this workshop. To help prepare students for telehealth practice in their future careers, educators should provide them with opportunities to practice and develop this critical skill set.

## Educational Objectives

By the end of this activity, learners will be able to:

1. Identify the unique challenges of telehealth with older adults.
2. Perform a telehealth clinical encounter with a patient over 65.
3. Feel comfortable with a telehealth encounter with a patient over 65.

## Introduction

The COVID-19 pandemic has had an enormous impact on the practice of medicine and medical education. In many cases, this has meant transitioning to virtual modalities to reduce the risk of viral exposure for health care providers, patients, and health professions students.

Prior to the pandemic, only around 4% of patients had used telehealth.^1,2^ During the pandemic, health systems nationwide rapidly converted outpatient clinic visits to virtual visits using telehealth technology, with some health systems reporting as much as a twentyfold increase in telehealth usage.^3–5^

Overall, the transition to telehealth has been well received, with both providers and patients reporting high levels of satisfaction.^6,7^ In fact, 66% of patients would like to continue having the option of telehealth visits in the future.^8^ Importantly, some centers have reported that nearly 70% of patients evaluated via telehealth were able to be managed at home, eliminating the possibility of viral exposures for patients and health care workers in these encounters.^5^

Additionally, increased usage of telehealth has persisted among older adults, even during periods when COVID-19 levels have been under control in the local area.^9^ As health care utilization in general is often higher among adults over the age of 65,^10,11^ it is important to develop the skills necessary to care for this population with telehealth.

Indeed, the surge of telehealth has brought the need for medical students to cultivate competency in this care modality to the fore.^12^ During the pandemic, medical students at all levels had their learning activities taken online.^13^ With the goal of preserving authentic patient experiences for learners, some programs have involved medical students in telehealth encounters or developed telehealth electives for students.^14,15^

Historically, opportunities for telehealth learning and instruction in traditional medical education have been limited. While the current generation of medical students can be considered digital natives, providing care with telehealth comes with unique challenges, including communicating empathy, navigating technical difficulties, and obtaining a physical exam.^16^ Furthermore, as more older adults participate in telehealth, the additional challenges of sensory and cognitive impairments, medical complexity, multimorbidity, and inclusion of family and/or caregivers must also be addressed.

Therefore, students would benefit from opportunities to practice telehealth encounters with older adults and develop their webside manner, preferentially through experiential learning, in which learning occurs through concrete experiences, reflection-observation, abstract conceptualization, and active experimentation in a low-stakes setting.^17^ Moreover, a formative activity in a low-stakes setting would likely best prepare students for higher-stakes clinical encounters with older adults.^18^

*MedEdPORTAL* has a wealth of publications pertaining to assessing and communicating with older adults.^19,20^ With increasing use of telehealth, there has been increasing development of telehealth educational materials on both general telehealth skills^21,22^ and care of older adults.^23^ Some incorporate cases with geriatric standardized patients (SPs).^24^ Others focus on more general topics and skills that are nonetheless high yield for those taking care of older adults.^25,26^ However, the resources in *MedEdPORTAL* and elsewhere in the literature do not challenge students to practice unifying their geriatrics communication skills, telehealth skills, and geriatric assessment skills in a simulated patient encounter. Our workshop adds to and expands upon the existing literature by challenging students to practice unifying all these skills in a simulated patient encounter. Here, we describe this workshop as well as the results from its implementation with rising clinical medical students over the years 2021–2022.

## Methods

This workshop was created in response to a local needs assessment by institutional leaders indicating that our rising third-year medical students had not received formal training on telemedicine despite a high likelihood they would participate in telemedicine during their clinical years. Author Lindsay A. Wilson, a geriatrician and clinician educator with experience developing, implementing, and evaluating geriatrics curricula utilizing SPs, codirected a course, the Transition to Application Phase (TAP), that could accommodate this curricular addition. This weeklong course for rising third-year medical students prepared them for their upcoming clinical rotations. The workshop was created under Dr. Wilson's leadership using the framework of experiential learning, in which learning occurs through concrete experiences, reflection-observation, abstract conceptualization, and active experimentation.^17^ To promote experiential learning in the workshop, students participated in and observed their peers in a simulated telehealth encounter with an SP (preferably an older adult), debriefed about their experience, and received immediate feedback. Students were observed and supported by a facilitator, a physician with expertise and/or interest in geriatric medicine. This exercise provided a safe learning environment by allowing students to practice their telehealth skills in a formative, low-stakes setting.^18^

The workshop was presented as part of the TAP course three times with either the entire cohort of TAP students or those students starting on their outpatient medicine rotation, a variation that resulted from changes in the availability of SPs and facilitators. The entire 90-minute workshop was carried out virtually using Zoom. In order to participate, facilitators and SPs were expected to have access to and experience with both computers and Zoom, including breakout rooms.

Learners first completed an electronic presurvey (Appendix A). Then Dr. Wilson presented a 20- to 30-minute didactic session (Appendix B), which outlined key tasks for a geriatric telemedicine visit such as assessing for sensory and cognitive impairments, involving caregivers, setting the visit agenda, triaging multiple clinical concerns, performing a geriatric review of systems, reconciling medications, counseling the patient, and confirming understanding with teach-back. The presentation also included instructions for the SP interactions.

Next, learners were divided into small groups of two to five students (based on availability of SPs and facilitators), one facilitator, and one SP for the SP activity. As each group needed an SP-facilitator dyad, the size of the student groups varied based on the availability of these dyads, which fluctuated over the three sessions due to resource constraints. Prior to the activity, facilitators and SPs received a guide for their roles (Appendix C) for review. The guide included recommendations on how to divide tasks for the visit between students, the suggested timeline for the session, the patient case background, and guidance on debriefing and providing feedback to learners. Facilitators and SPs also had the option of attending a brief training session prior to the workshop to ask questions and discuss the workshop format. Most of the SPs had experience working with medical students and participating in similar workshops. Older adult SPs were requested; if they were not available, an SP of middle age substituted and acted the role of the older adult.

Learners received the student guide (Appendix D), which provided patient case background in the form of the clinic note from a previous visit and the medication list. On completion of the SP visit, which lasted an average of 30 minutes, students debriefed and received feedback from the facilitator and the SP for about 20 minutes. This was followed by completion of an electronic postsurvey (Appendix A).

The pre- and postsurveys asked learners to rate their level of comfort using telemedicine and caring for people over the age of 65 on a 5-point Likert-type scale (1 = *very uncomfortable,* 5 = *very comfortable*). Difference in level of comfort was evaluated with a nonparametric Wilcoxon rank sum test. SAS version 9.4 (SAS Institute) was used for statistical analysis.

Qualitatively, students were asked three open-ended questions to better understand their initial concerns about using telemedicine with older adults and provide more expansive feedback about the workshop's usefulness and appeal. Students were specifically asked what they would change about the workshop in future iterations to guide continuous quality improvement. Responses were reviewed for major themes and considered in relation to the quantitative results.

No participant identifiers were collected, which precluded paired comparison analyses. Institutional review board exemption (IRB# 20-2862) was obtained.

## Results

This workshop was presented to 58 third-year medical students. Facilitators included internal and family medicine residents, geriatric medicine fellows, and faculty physicians from internal and family medicine.

Learners completed surveys before and after the activity (presurvey = 58, postsurvey = 40). Many students did not complete the postsurvey as it was not required for the course. Fifty-two percent of students reported prior experience with telemedicine. Learners reported statistically significant increases in comfort using telemedicine (presurvey = 3.1, postsurvey = 3.9, *p* < .001), using telemedicine for patients ≥65 years of age (presurvey = 2.8, postsurvey = 3.9, *p* < .001), interviewing patients over telemedicine (presurvey = 3.4, postsurvey = 4.0, *p* < .001), interviewing patients ≥65 years of age over telemedicine (presurvey = 3.0, postsurvey = 4.0, *p* < .001), managing patients over telemedicine (presurvey = 2.7, postsurvey = 3.7, *p* < .001), and managing patients ≥65 years of age over telemedicine (presurvey = 2.5, postsurvey = 3.7, *p* < .001) after completing the workshop.

Qualitatively, learners expressed concerns about older adults’ ability to use technology and potential vision and hearing deficits impeding communication. Students reported a positive experience with the workshop, specifically the opportunity to practice delivering care in the virtual setting. For example, when asked what they liked about the workshop, one student shared, “Practice with a real patient!” Another responded, “Practice reviewing topics to cover with patients >65yo (ADLs [activities of daily living], IADLs [instrumental activities of daily living], etc).”

## Discussion

In the COVID-19 era, telehealth emerged as an important tool for continuing to provide care during a global health disaster. Having proven its value, telehealth will remain part of the health care landscape. As such, it is necessary to prepare learners to utilize telehealth in their future careers.

This workshop helped medical students develop their skills as telehealth providers by requiring them to perform a telehealth clinic visit with an older adult who may have been developing cognitive impairment and needed close attention to medication reconciliation. Importantly, the workshop integrated principles of geriatric care, specifically cognitive assessment, medication management, and functional assessment, preparing students to address the unique aspects of working with this special population via telemedicine.

The goal of the workshop was to provide students with an opportunity to practice a telehealth visit with a geriatric patient in a safe learning environment. The evaluation of this program showed significant increases in learners’ level of comfort with using telemedicine to evaluate and manage geriatric patients.

Among the lessons we learned is that medical students appreciated the opportunity to practice their telehealth and clinical skills in this format prior to starting their clinical training. The workshop structure was remarkably adaptable to the availability of the facilitators and SPs, with all groups reporting positive experiences regardless of group size. We did not exceed five students in a group in order to allow each student to actively participate. If only two students were in a group, the assignments were adjusted accordingly. For example, one student would complete tasks A and B, the other student would complete tasks C and D, and they would both provide feedback to each other (task E). The smaller groups allowed for even more participation on the part of the students, which may have enhanced learning; however, our evaluation did not investigate the impact of group size on learning or overall experience, which could be an area for future research.

Facilitators with a high level of interest and experience in geriatrics and undergraduate medical education were selected. Depending on availability, facilitators included resident physicians from family medicine and internal medicine, geriatric medicine fellows, and faculty from general internal medicine, family medicine, and geriatric medicine. Informal discussions with facilitators indicated that they had a positive experience regardless of level of training.

The SPs were older adults or middle-age adults acting as older adults if needed. Both the facilitators and the SPs were encouraged to be flexible and adapt if any unexpected situations arose (such as an SP going off script) and to make the session fun and low stakes. One recommendation going forward is that facilitators and SPs should appreciate that rising clinical medical students have varying experience with geriatrics, telemedicine, and clinical medicine in general. Overly critical feedback is likely to negatively impact students’ experiences and should be discouraged.

Our approach to evaluation focused on change in student comfort level with telemedicine. In the future, in addition to comfort level, it may be helpful to further assess students’ perceptions of telehealth as well as measure growth in their skills. SPs, facilitators, or other observers could use a checklist to evaluate students’ ability to complete key elements. This type of assessment was beyond the scope of this workshop but could be added to future iterations. It may also be helpful to assess the facilitator and SP experience with this activity prior to further expansion.

While this workshop was developed primarily for medical students, it could easily be adapted for other health professions students or as an interprofessional learning activity. Similarly, the main focus of this activity was on communication strategies for history gathering and counseling of geriatric patients via telemedicine rather than on a telemedicine physical exam. A physical exam—for example, of the knee—could be added as a requirement.

At this point, telehealth is widespread, and medical students will be expected to demonstrate competence with it. For future directions, programs may move from low-stakes formative activities like this one to more formal assessment of telehealth skills such as a telehealth OSCE. To help prepare students for telehealth practice in their careers, educators should strive to provide them with opportunities to practice and develop this critical skill set.

## Appendices


Pre- and Postsurvey.docxGeriatric Telehealth Didactic.pptxFacilitator and SP Guide.docxStudent Guide.docx
*All appendices are peer reviewed as integral parts of the Original Publication.*

## References

[1] Welch BM, Harvey J, O'Connell NS, McElligott JT. Patient preferences for direct-to-consumer telemedicine services: a nationwide survey. BMC Health Serv Res. 2017;17:784. 10.1186/s12913-017-2744-8 29183372 PMC5704580

[2] Fischer SH, Ray KN, Mehrotra A, Bloom EL, Uscher-Pines L. Prevalence and characteristics of telehealth utilization in the United States. JAMA Netw Open. 2020;3(10):e2022302. 10.1001/jamanetworkopen.2020.22302 33104208 PMC7588937

[3] Cantor JH, McBain RK, Pera MF, Bravata DM, Whaley CM. Who is (and is not) receiving telemedicine care during the COVID-19 pandemic. Am J Prev Med. 2021;61(3):434–438. 10.1016/j.amepre.2021.01.030 33781622 PMC7936544

[4] Mann DM, Chen J, Chunara R, Testa PA, Nov O. COVID-19 transforms health care through telemedicine: evidence from the field. J Am Med Inform Assoc. 2020;27(7):1132–1135. 10.1093/jamia/ocaa072 32324855 PMC7188161

[5] Koonin LM, Hoots B, Tsang CA, et al. Trends in the use of telehealth during the emergence of the COVID-19 pandemic—United States, January–March 2020. MMWR Morb Mortal Wkly Rep. 2020;69(43):1595–1599. 10.15585/mmwr.mm6943a3 33119561 PMC7641006

[6] Bate NJ, Xu SC, Pacilli M, Roberts LJ, Kimber C, Nataraja RM. Effect of the COVID-19 induced phase of massive telehealth uptake on end-user satisfaction. Intern Med J. 2021;51(2):206–214. 10.1111/imj.15222 33631844 PMC8014045

[7] Dobrusin A, Hawa F, Gladshteyn M, et al. Gastroenterologists and patients report high satisfaction rates with telehealth services during the novel coronavirus 2019 pandemic. Clin Gastroenterol Hepatol. 2020;18(11):2393–2397.e2. 10.1016/j.cgh.2020.07.014 32663521 PMC7352104

[8] Predmore ZS, Roth E, Breslau J, Fischer SH, Uscher-Pines L. Assessment of patient preferences for telehealth in post–COVID-19 pandemic health care. JAMA Netw Open. 2021;4(12):e2136405. 10.1001/jamanetworkopen.2021.36405 34851400 PMC8637257

[9] Tan LF, Seetharaman SK, Yip WA. Telehealth utilisation amongst older adults during a pandemic year. Geriatr Gerontol Int. 2021;21(5):439–440. 10.1111/ggi.14161 33786959 PMC8251494

[10] Cheng Y, Goodin AJ, Pahor M, Manini T, Brown JD. Healthcare utilization and physical functioning in older adults in the United States. J Am Geriatr Soc. 2020;68(2):266–271. 10.1111/jgs.16260 31755551

[11] Zayas CE, He Z, Yuan J, et al. Examining healthcare utilization patterns of elderly middle-aged adults in the United States. In: Proceedings of the Twenty-Ninth International Florida Artificial Intelligence Research Society Conference. Association for the Advancement of Artificial Intelligence; 2016:361–366. PMC4946167 27430035

[12] Association of American Medical Colleges. Telehealth Competencies Across the Learning Continuum. Association of American Medical Colleges; 2021. Accessed July 28, 2023. https://store.aamc.org/downloadable/download/sample/sample_id/412

[13] Rose S. Medical student education in the time of COVID-19. JAMA. 2020;323(21):2131–2132. 10.1001/jama.2020.5227 32232420

[14] Lucey CR, Johnston SC. The transformational effects of COVID-19 on medical education. JAMA. 2020;324(11):1033–1034. 10.1001/jama.2020.14136 32857137

[15] Weber AM, Dua A, Chang K, et al. An outpatient telehealth elective for displaced clinical learners during the COVID-19 pandemic. BMC Med Educ. 2021;21:174. 10.1186/s12909-021-02604-z 33743676 PMC7980791

[16] Iancu AM, Kemp MT, Alam HB. Unmuting medical students’ education: utilizing telemedicine during the COVID-19 pandemic and beyond. J Med Internet Res. 2020;22(7):e19667. 10.2196/19667 32614776 PMC7400037

[17] Kolb DA. Experiential Learning: Experience as the Source of Learning and Development. 2nd ed. Pearson Education; 2015.

[18] Young JE, Williamson MI, Egan TG. Students’ reflections on the relationships between safe learning environments, learning challenge and positive experiences of learning in a simulated GP clinic. Adv Health Sci Educ Theory Pract. 2016;21(1):63–77. 10.1007/s10459-015-9611-3 25952645

[19] Agness-Whittaker CF, Macedo L. Aging, culture, and health communication: exploring personal cultural health beliefs and strategies to facilitate cross-cultural communication with older adults. MedEdPORTAL. 2016;12:10374. 10.15766/mep_2374-8265.10374

[20] Phillips SC, Hawley CE, Triantafylidis LK, Schwartz AW. Geriatrics 5Ms for primary care workshop. MedEdPORTAL. 2019;15:10814. 10.15766/mep_2374-8265.10814 31139733 PMC6489378

[21] Cornes S, Gelfand JM, Calton B. Foundational telemedicine workshop for first-year medical students developed during a pandemic. MedEdPORTAL. 2021;17:11171. 10.15766/mep_2374-8265.11171 34337148 PMC8282677

[22] Mulcare M, Naik N, Greenwald P, et al. Advanced communication and examination skills in telemedicine: a structured simulation-based course for medical students. MedEdPORTAL. 2020;16:11047. 10.15766/mep_2374-8265.11047 33365390 PMC7751329

[23] Goldberg GR, Solis G, John JT, Olvet DM, Kranz KA. 4Ms for early learners: a skills-based geriatrics curriculum for second-year medical students. MedEdPORTAL. 2022;18:11264. 10.15766/mep_2374-8265.11264 35847421 PMC9237204

[24] Shortridge A, Steinheider B, Ciro C, Randall K, Costner-Lark A, Loving G. Simulating interprofessional geriatric patient care using telehealth: a team-based learning activity. MedEdPORTAL. 2016;12:10415. 10.15766/mep_2374-8265.10415 31008195 PMC6464453

[25] Afonso N, Kelekar A, Alangaden A. “I have a cough”: an interactive virtual respiratory case-based module. MedEdPORTAL. 2020;16:11058. 10.15766/mep_2374-8265.11058 33365392 PMC7751326

[26] Cantone RE, Palmer R, Dodson LG, Biagioli FE. Insomnia telemedicine OSCE (teleOSCE): a simulated standardized patient video-visit case for clerkship students. MedEdPORTAL. 2019;15:10867. 10.15766/mep_2374-8265.10867 32051850 PMC7012306

