# Supplementary material for: Geriatric Telehealth: A Standardized Patient Case for Medical Students
Source: MedEdPORTAL. 2023 Sep 12;19:11345. doi: 10.15766/mep_2374-8265.11345 (PMC10495538; doi:10.15766/mep_2374-8265.11345)
Supplement: Supplementary file 1 — Pre- and Postsurvey.docxGeriatric Telehealth Didactic.pptxFacilitator and SP Guide.docxStudent Guide.docx [file mep_2374-8265.11345-s001.zip › A. Pre- and Postsurvey.docx]

***Telemedicine*** *may be defined as the practice of providing solely medical services at a distance through platforms such as video conferencing programs (i.e., Zoom, Doxy.me, etc.) and phone calls*.

Pre-Workshop Survey Questions:

1. Have you had experience with telemedicine?

- Yes
- No

1. How comfortable are you with using telemedicine?

- Very Comfortable
- Comfortable
- Uncomfortable
- Very Uncomfortable
- Unknown

1. How comfortable are you with using telemedicine for patients > 65 years old?

- Very Comfortable
- Comfortable
- Uncomfortable
- Very Uncomfortable
- Unknown

1. How comfortable are you interviewing a patient over telemedicine?

- Very Comfortable
- Comfortable
- Uncomfortable
- Very Uncomfortable
- Unknown

1. How comfortable are you interviewing a patient > 65 years old over telemedicine?

- Very Comfortable
- Comfortable
- Uncomfortable
- Very Uncomfortable
- Unknown

1. How comfortable are you managing a patient over telemedicine?

- Very Comfortable
- Comfortable
- Uncomfortable
- Very Uncomfortable
- Unknown

1. How comfortable are you managing a patient > 65 years old over telemedicine?

- Very Comfortable
- Comfortable
- Uncomfortable
- Very Uncomfortable
- Unknown

1. What are your concerns about using telemedicine with patients > 65 yrs old?

- Very Comfortable
- Comfortable
- Uncomfortable
- Very uncomfortable
- Unknown

1. What are your concerns about using telemedicine with patients > 65 years old?

Post-Workshop Survey Questions:

1. Have you had experience with telemedicine?

- Yes
- No

1. How comfortable are you with using telemedicine?

- Very Comfortable
- Comfortable
- Uncomfortable
- Very Uncomfortable
- Unknown

1. How comfortable are you with using telemedicine for patients > 65 years old?

- Very Comfortable
- Comfortable
- Uncomfortable
- Very Uncomfortable
- Unknown

1. How comfortable are you interviewing a patient over telemedicine?

- Very Comfortable
- Comfortable
- Uncomfortable
- Very Uncomfortable
- Unknown

1. How comfortable are you interviewing a patient > 65 years old over telemedicine?

- Very Comfortable
- Comfortable
- Uncomfortable
- Very Uncomfortable
- Unknown

1. How comfortable are you managing a patient over telemedicine?

- Very Comfortable
- Comfortable
- Uncomfortable
- Very Uncomfortable
- Unknown

1. How comfortable are you managing a patient > 65 years old over telemedicine?

- Very Comfortable
- Comfortable
- Uncomfortable
- Very Uncomfortable
- Unknown

1. What are your concerns about using telemedicine with patients > 65 yrs old?

- Very Comfortable
- Comfortable
- Uncomfortable
- Very Uncomfortable
- Unknown

1. What was most useful about this workshop?
2. What did you not like about this workshop?
3. What would you change about this workshop in the future?
