# Supplementary material for: Geriatric Telehealth: A Standardized Patient Case for Medical Students
Source: MedEdPORTAL. 2023 Sep 12;19:11345. doi: 10.15766/mep_2374-8265.11345 (PMC10495538; doi:10.15766/mep_2374-8265.11345)
Supplement: Supplementary file 1 — Pre- and Postsurvey.docxGeriatric Telehealth Didactic.pptxFacilitator and SP Guide.docxStudent Guide.docx [file mep_2374-8265.11345-s001.zip › B. Geriatric Telehealth Didactic.pptx]

## Slide 1
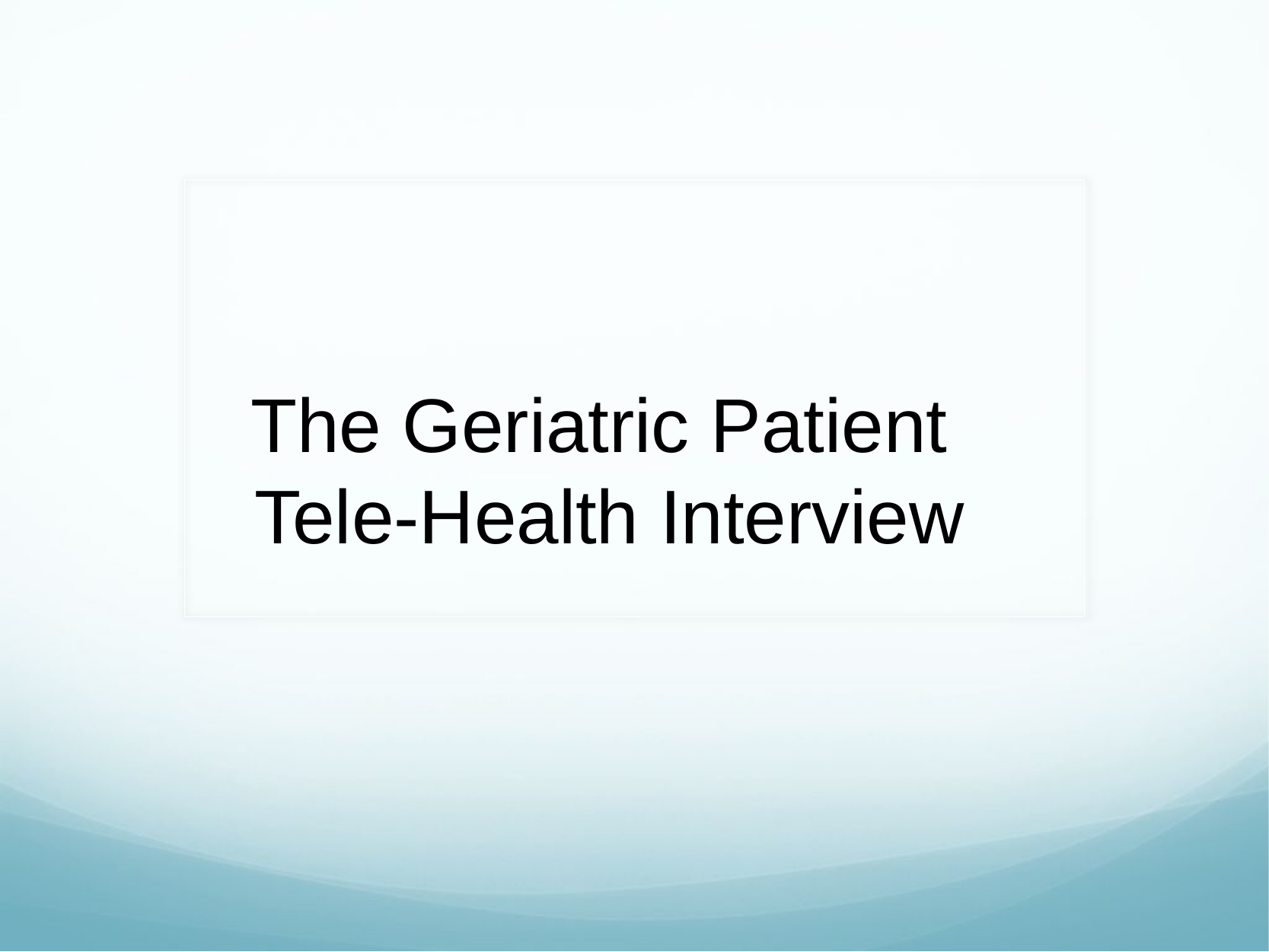

# The Geriatric Patient Tele-Health Interview

## Slide 2
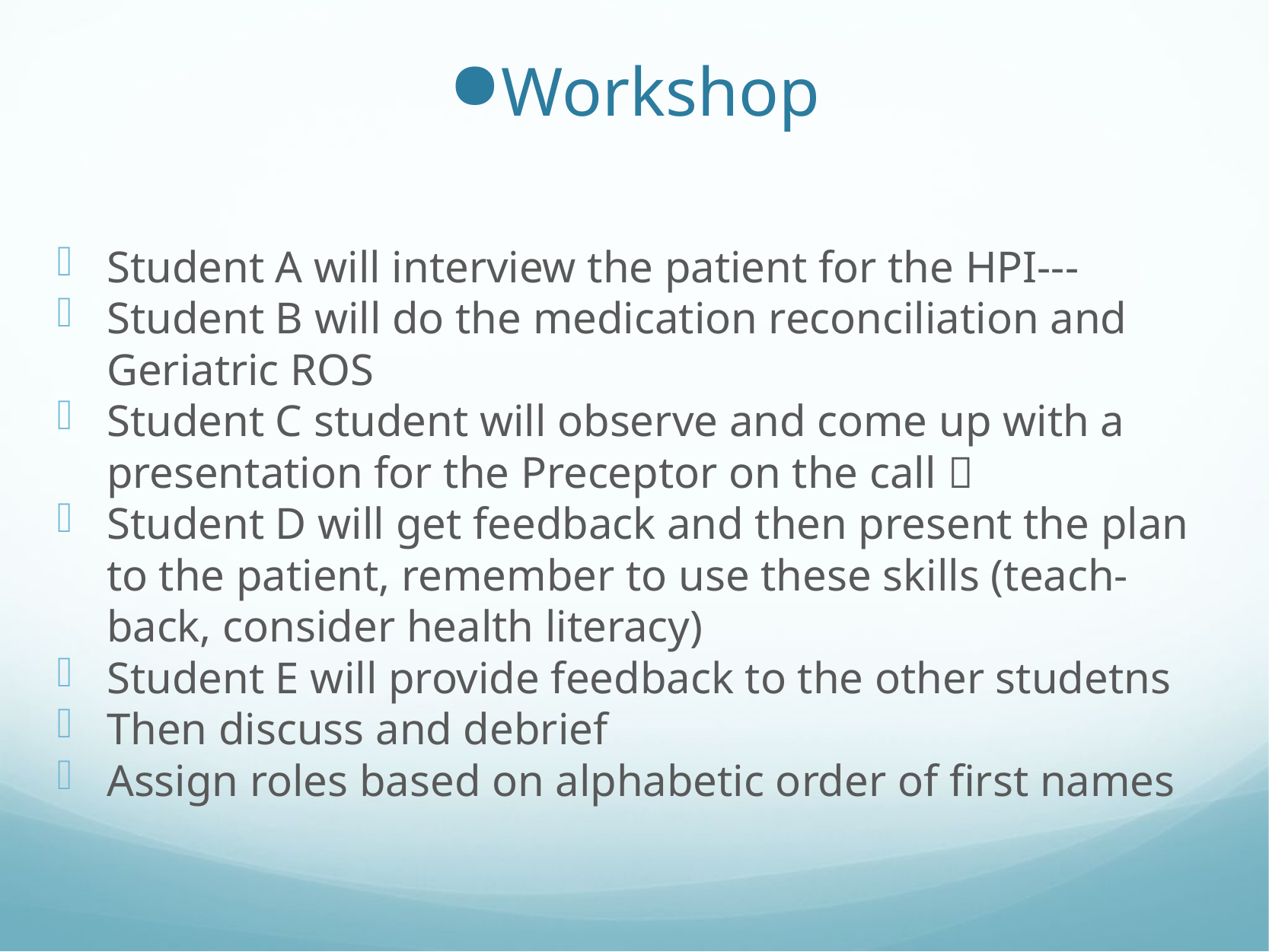

# Workshop
Student A will interview the patient for the HPI---
Student B will do the medication reconciliation and Geriatric ROS
Student C student will observe and come up with a presentation for the Preceptor on the call 
Student D will get feedback and then present the plan to the patient, remember to use these skills (teach-back, consider health literacy)
Student E will provide feedback to the other studetns
Then discuss and debrief
Assign roles based on alphabetic order of first names

## Slide 3
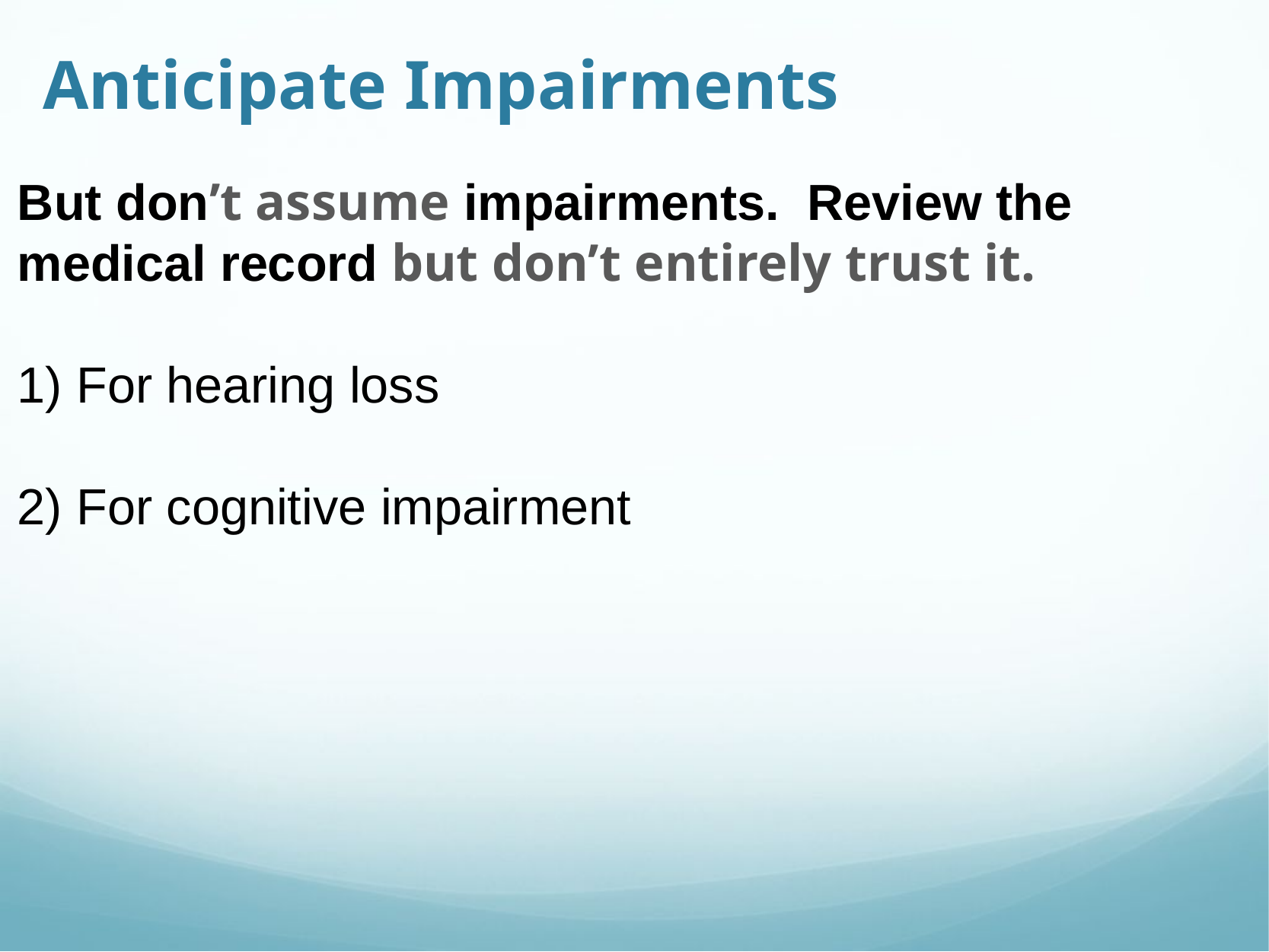

# Anticipate Impairments
But don’t assume impairments. Review the medical record but don’t entirely trust it.
1) For hearing loss
2) For cognitive impairment

## Slide 4
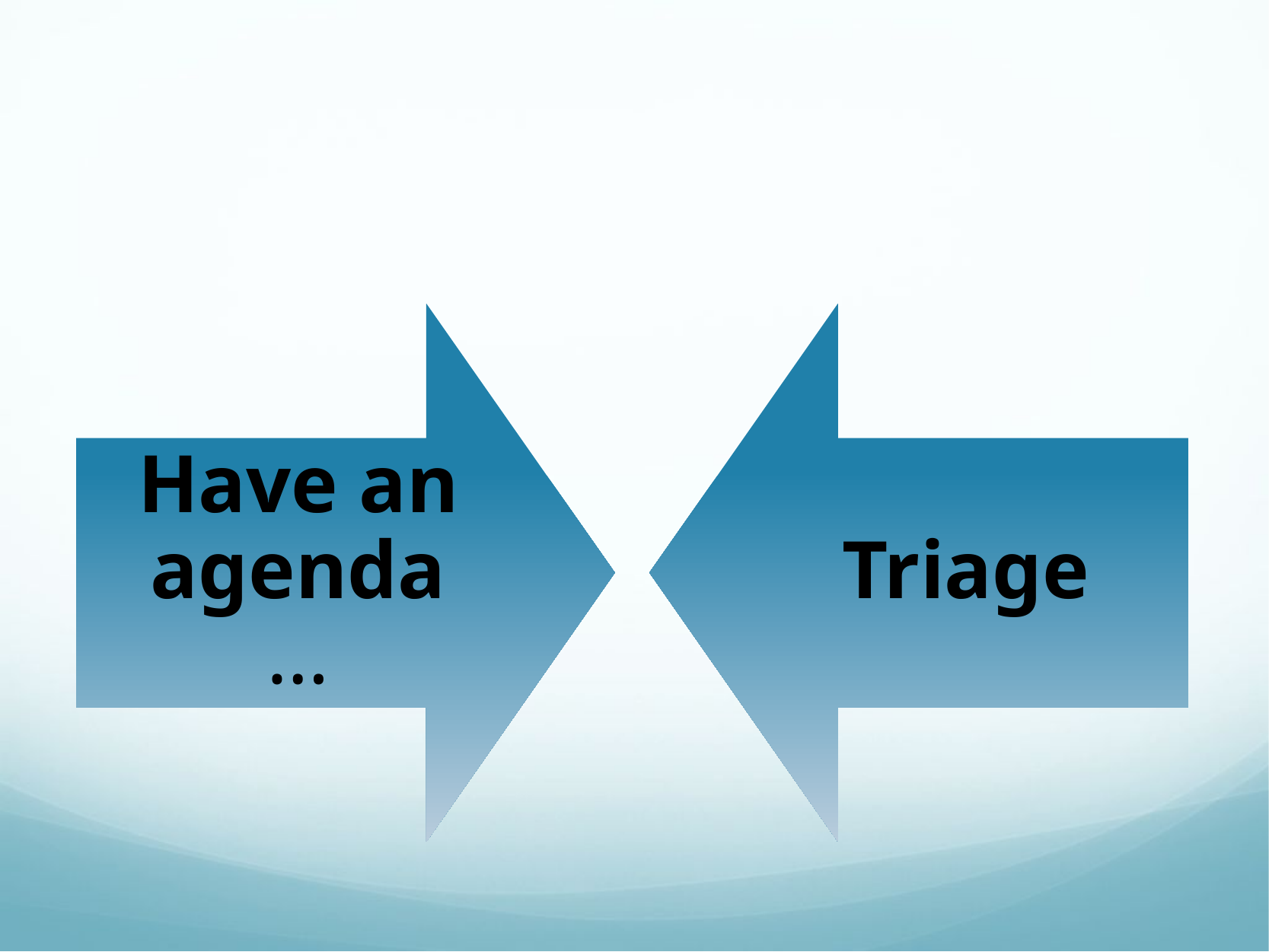

#

## Slide 5
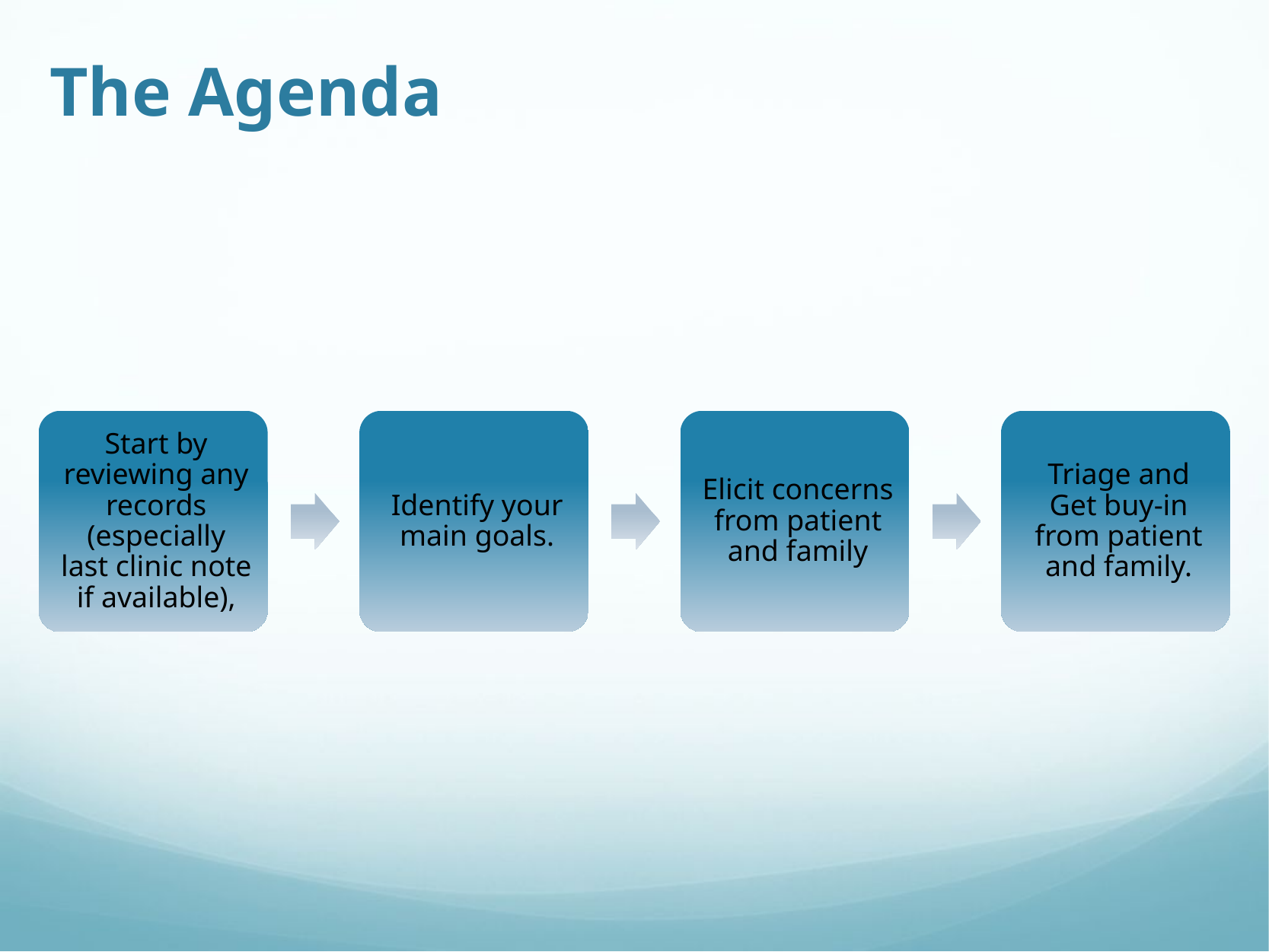

# The Agenda

## Slide 6
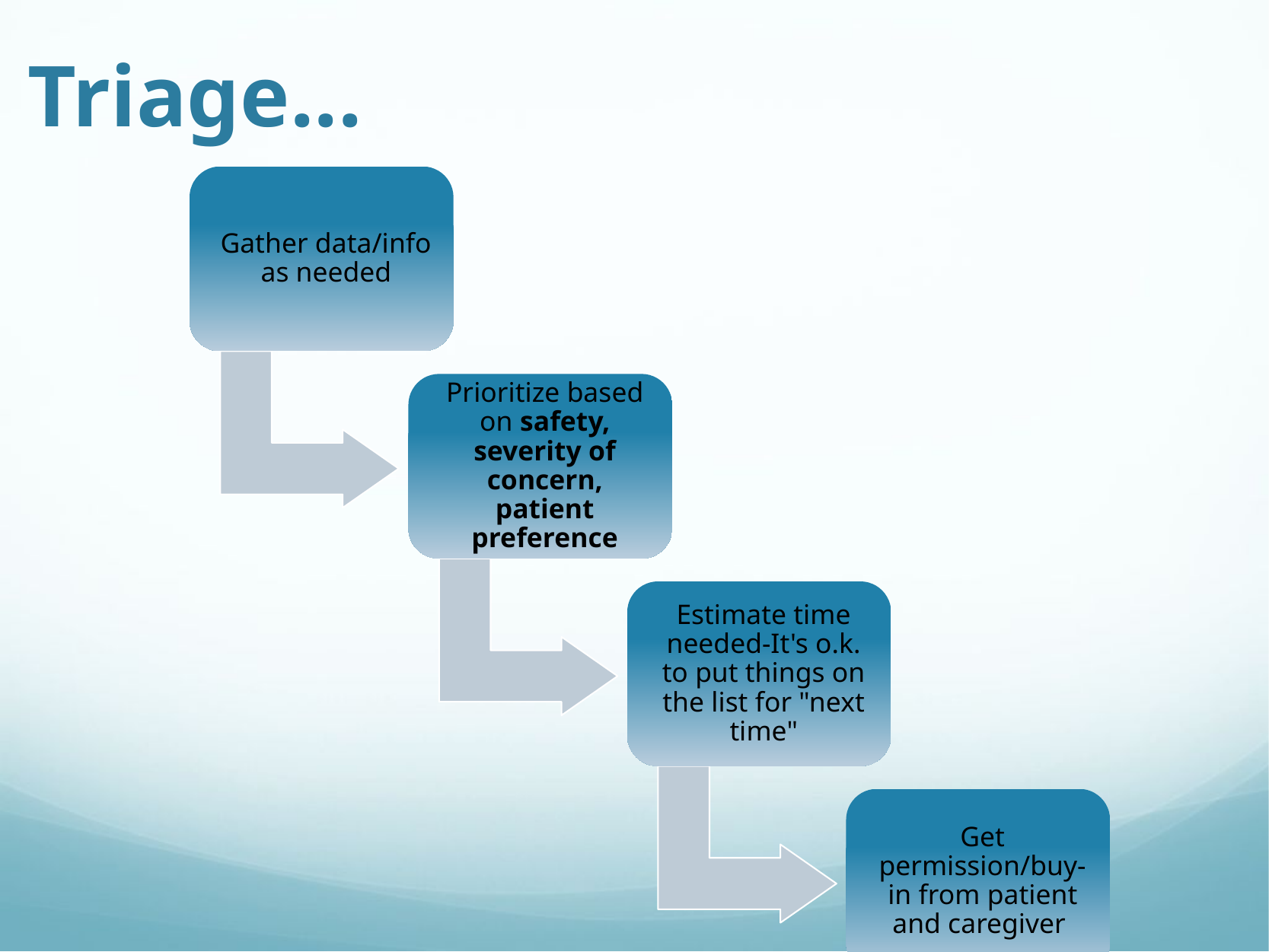

# Triage...

## Slide 7
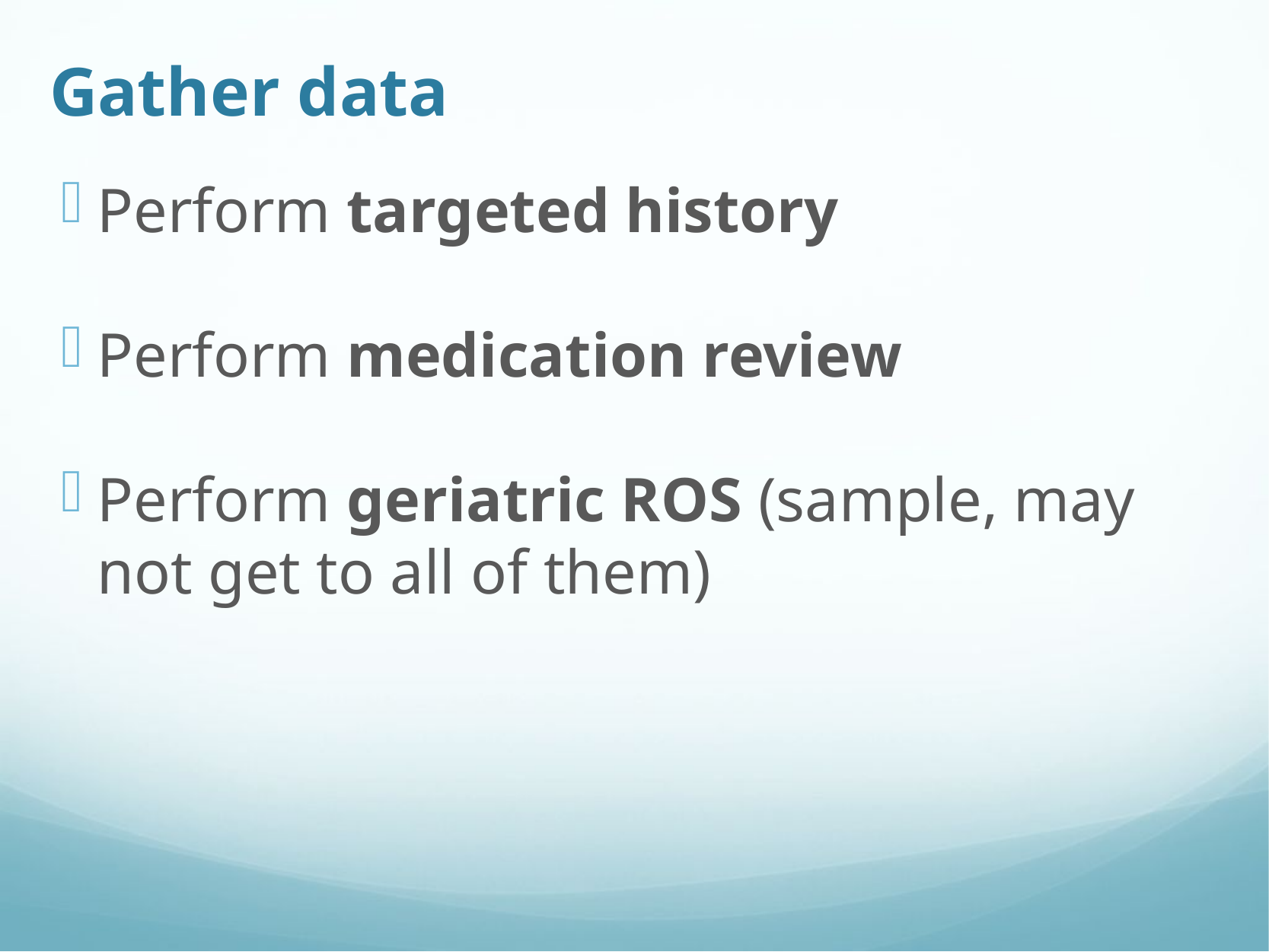

# Gather data
Perform targeted history
Perform medication review
Perform geriatric ROS (sample, may not get to all of them)

## Slide 8
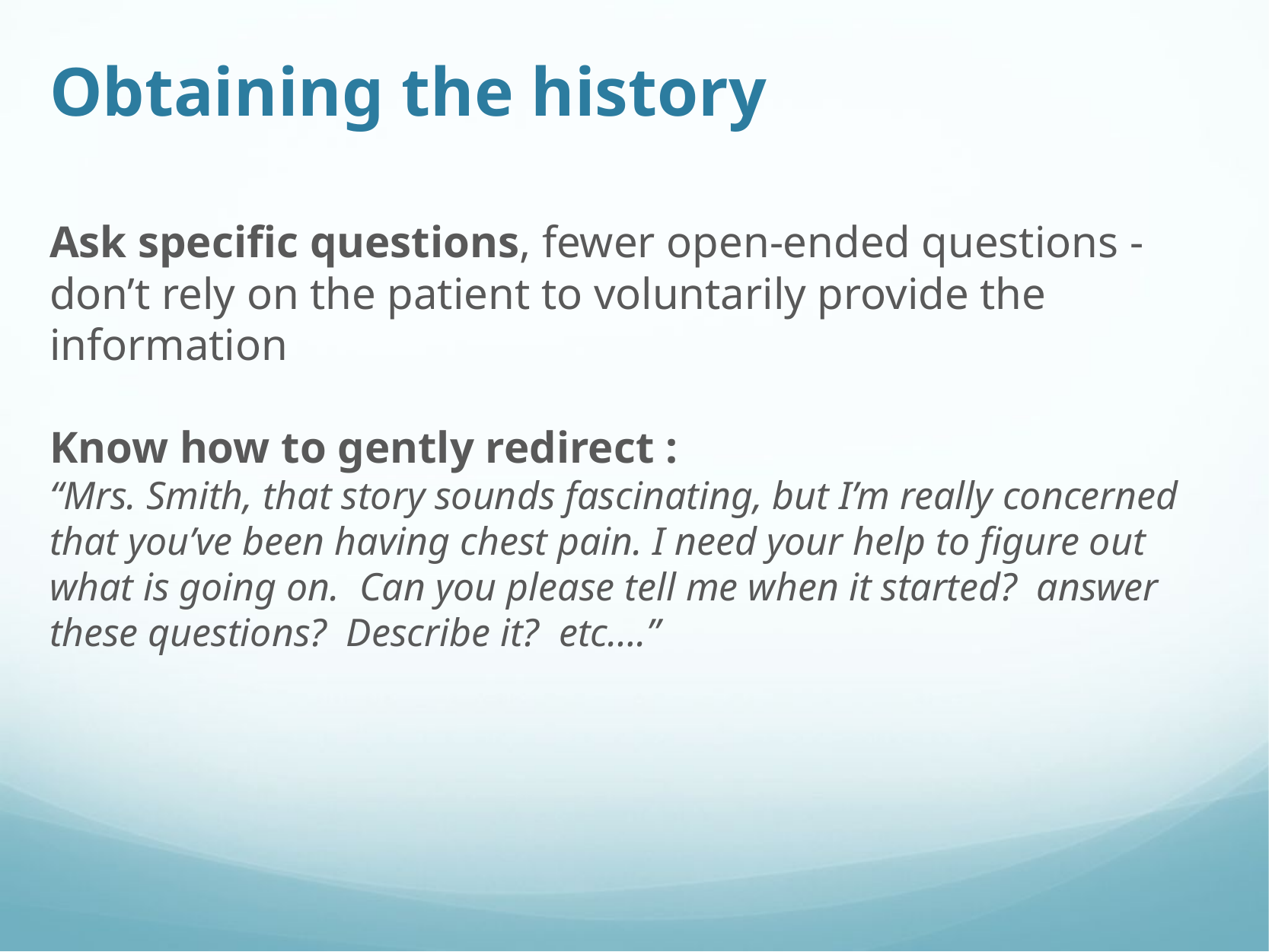

# Obtaining the history
Ask specific questions, fewer open-ended questions - don’t rely on the patient to voluntarily provide the information
Know how to gently redirect :
“Mrs. Smith, that story sounds fascinating, but I’m really concerned that you’ve been having chest pain. I need your help to figure out what is going on. Can you please tell me when it started? answer these questions? Describe it? etc….”

## Slide 9
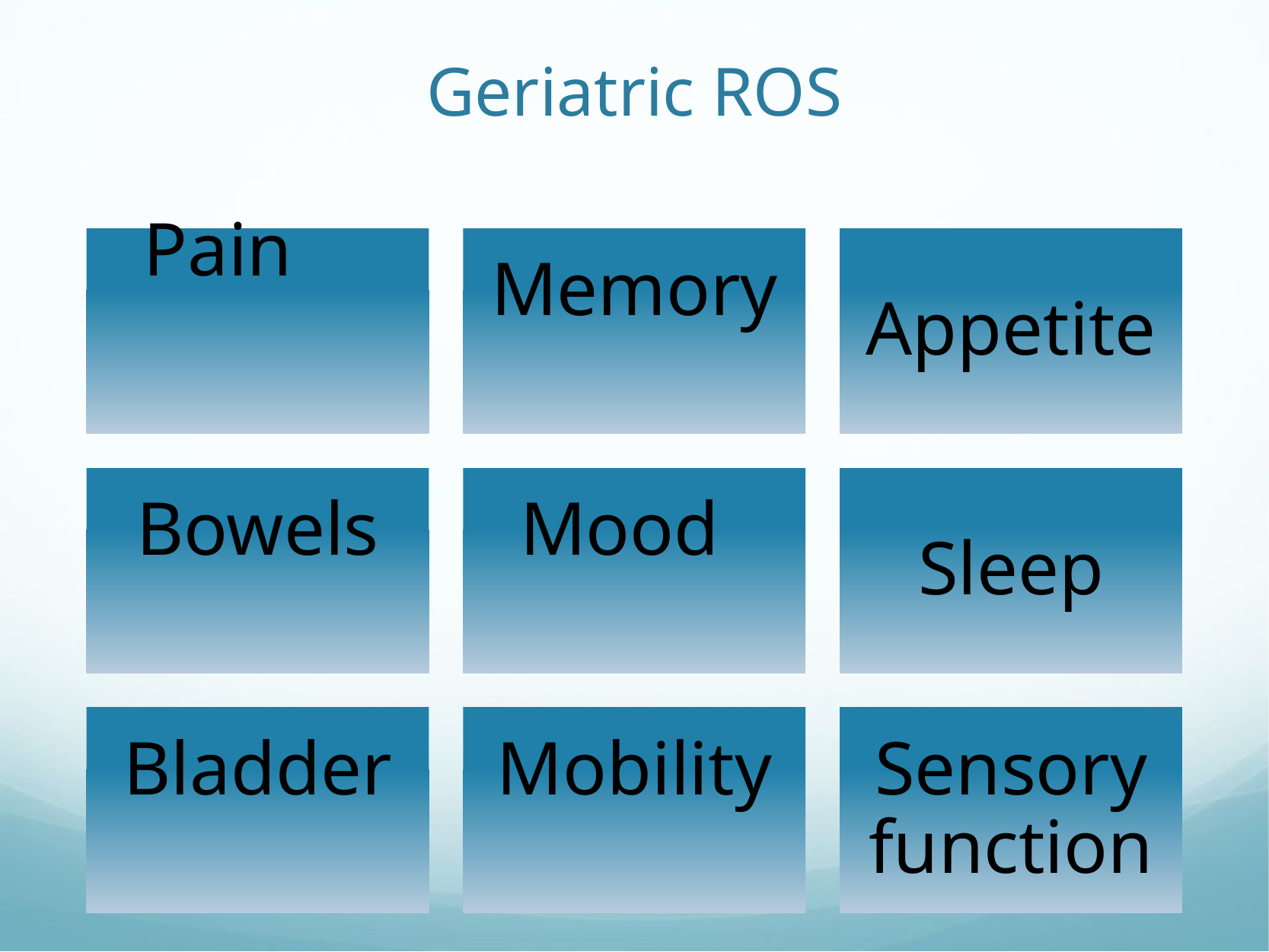

# Geriatric ROS

## Slide 10
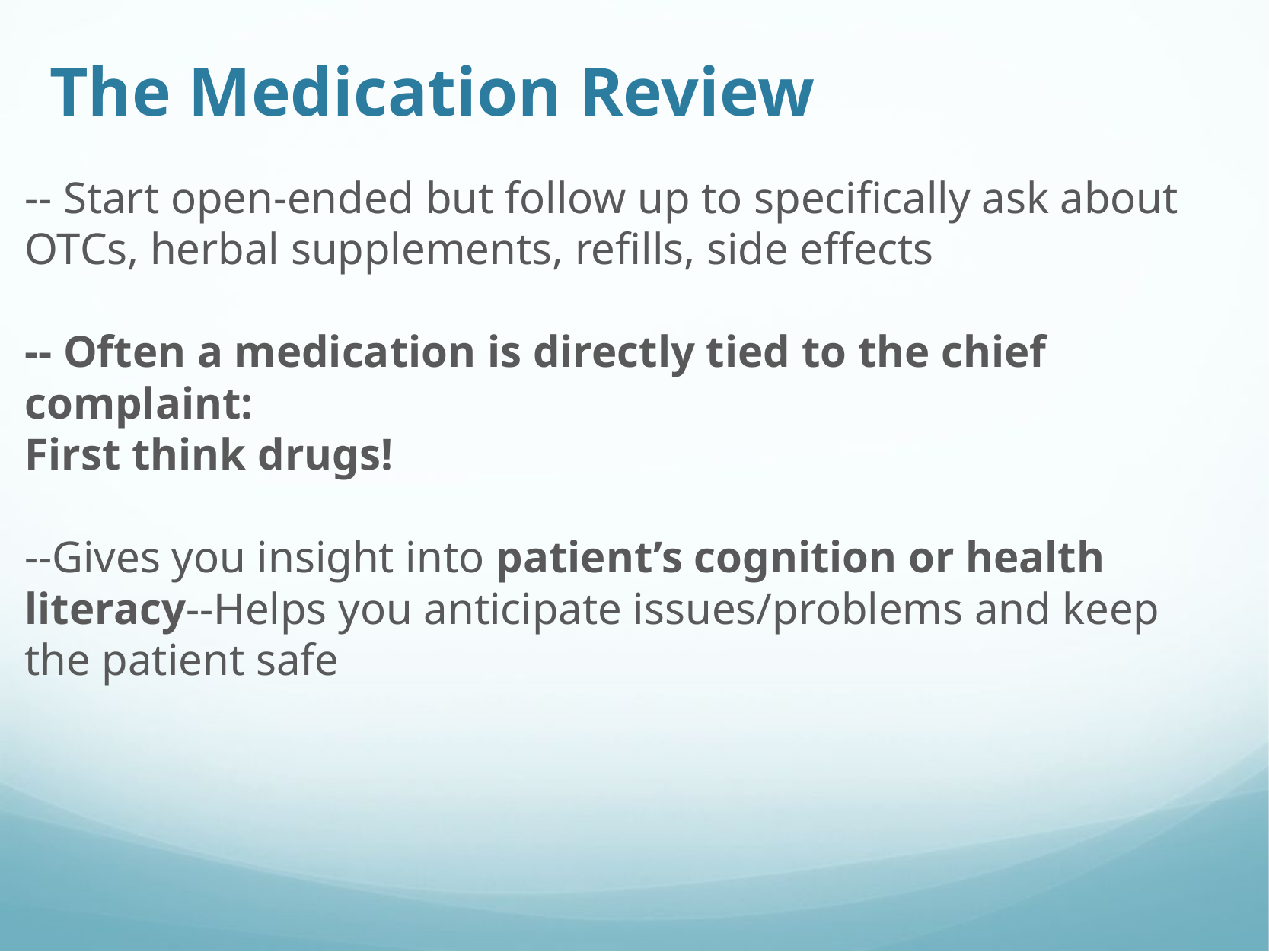

# The Medication Review
-- Start open-ended but follow up to specifically ask about OTCs, herbal supplements, refills, side effects
-- Often a medication is directly tied to the chief complaint:
First think drugs!
--Gives you insight into patient’s cognition or health literacy--Helps you anticipate issues/problems and keep the patient safe

## Slide 11
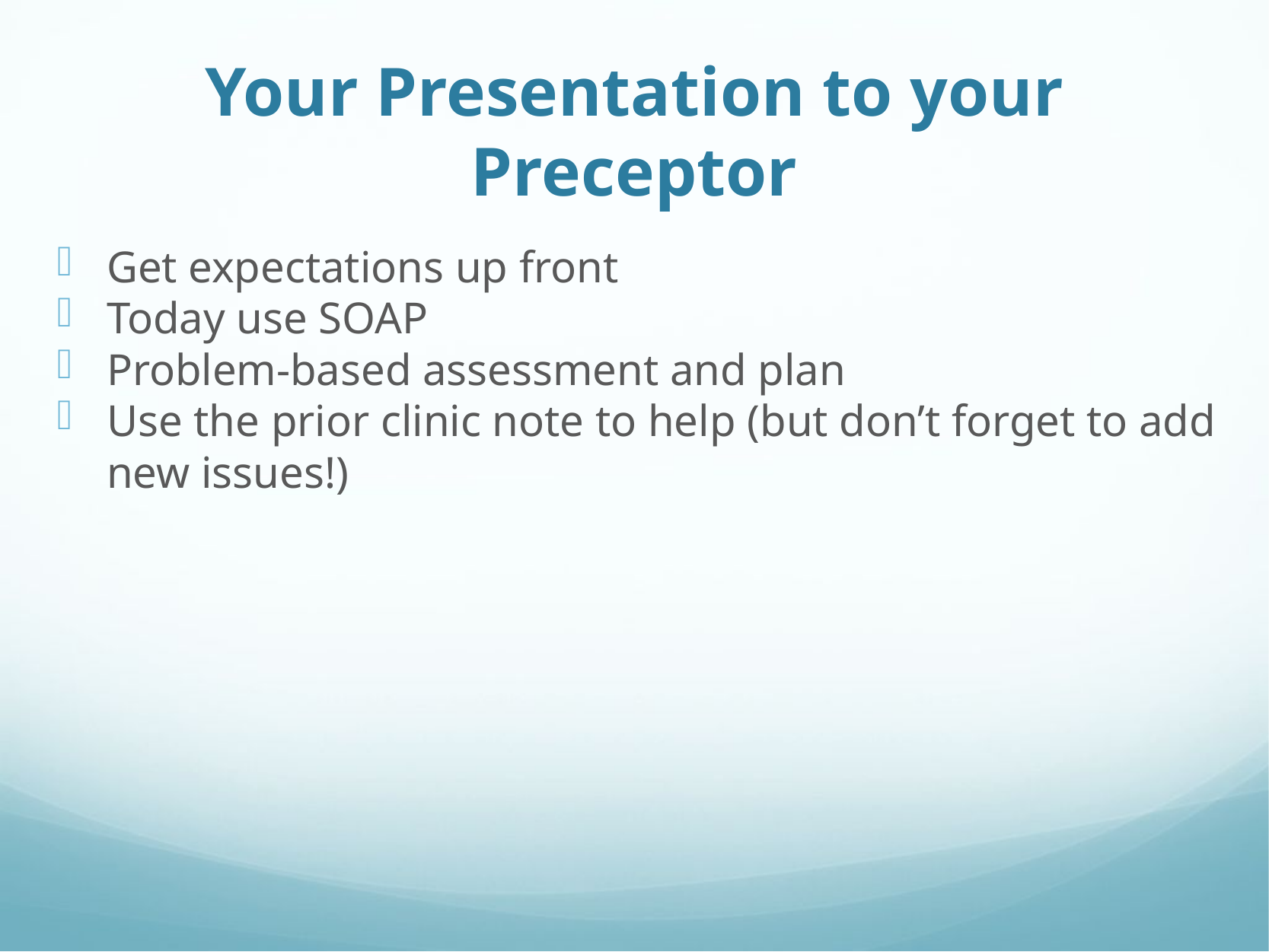

# Your Presentation to your Preceptor
Get expectations up front
Today use SOAP
Problem-based assessment and plan
Use the prior clinic note to help (but don’t forget to add new issues!)

## Slide 12
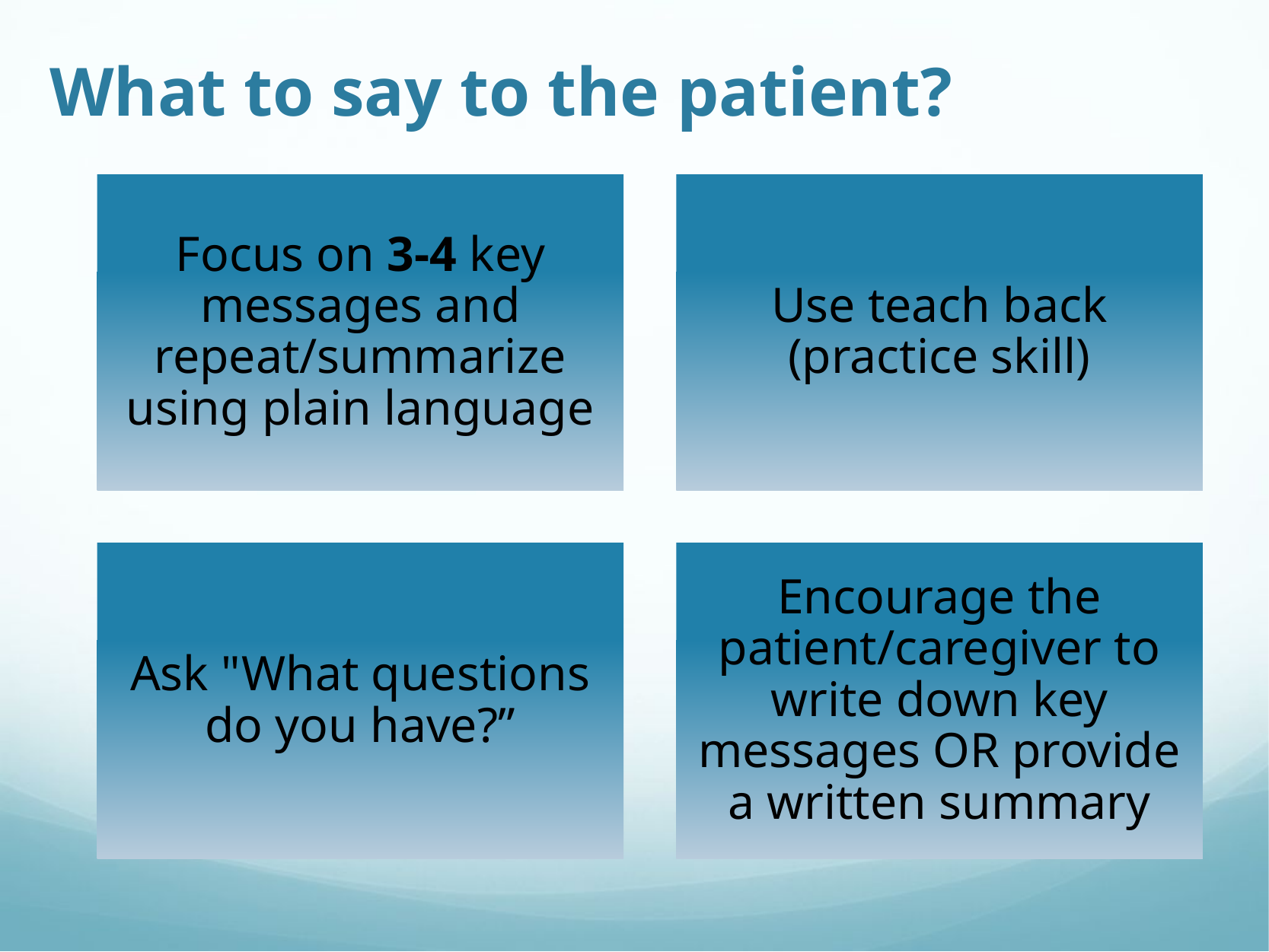

# What to say to the patient?

## Slide 13
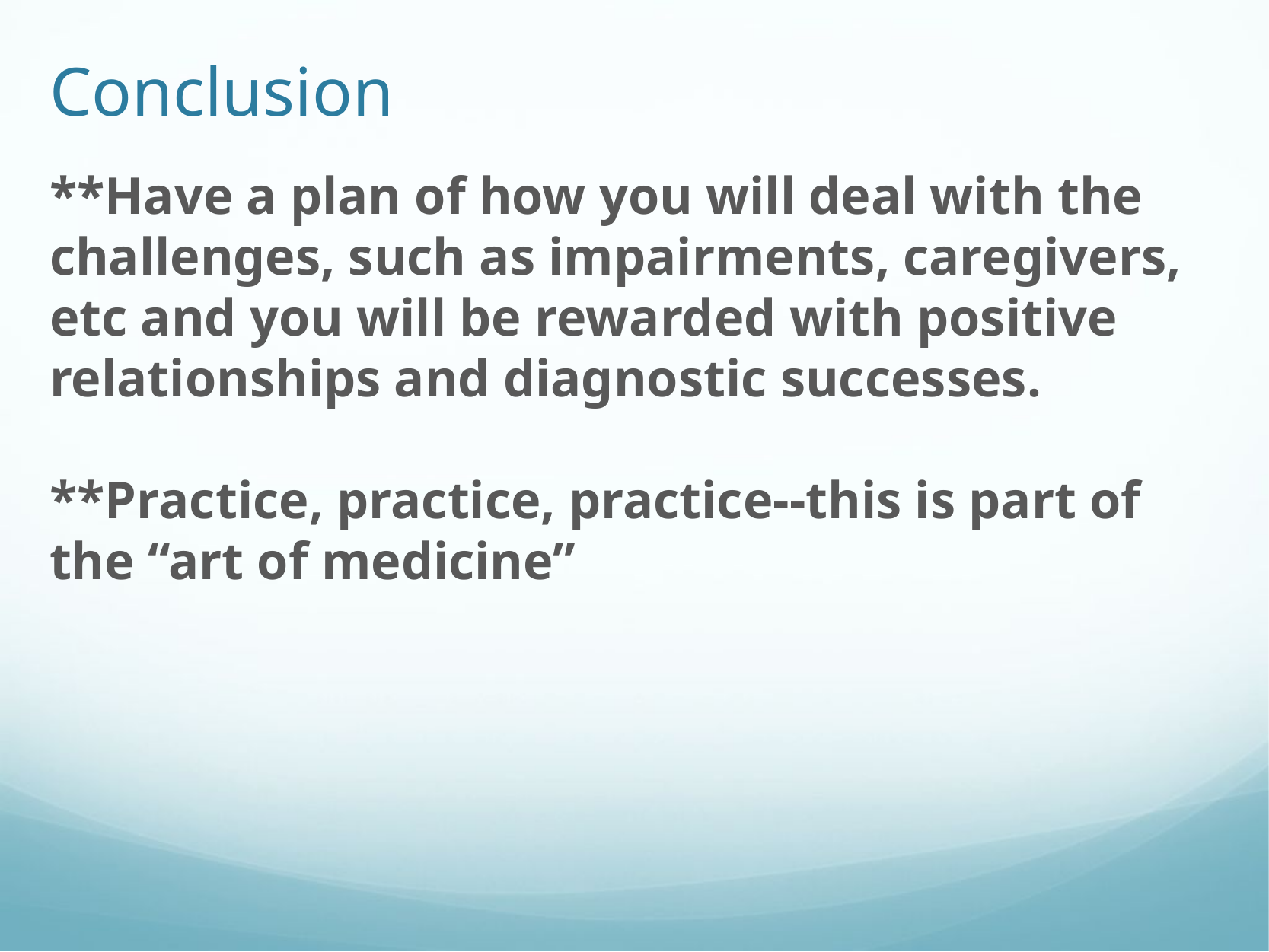

# Conclusion
**Have a plan of how you will deal with the challenges, such as impairments, caregivers, etc and you will be rewarded with positive relationships and diagnostic successes.
**Practice, practice, practice--this is part of the “art of medicine”

## Slide 14
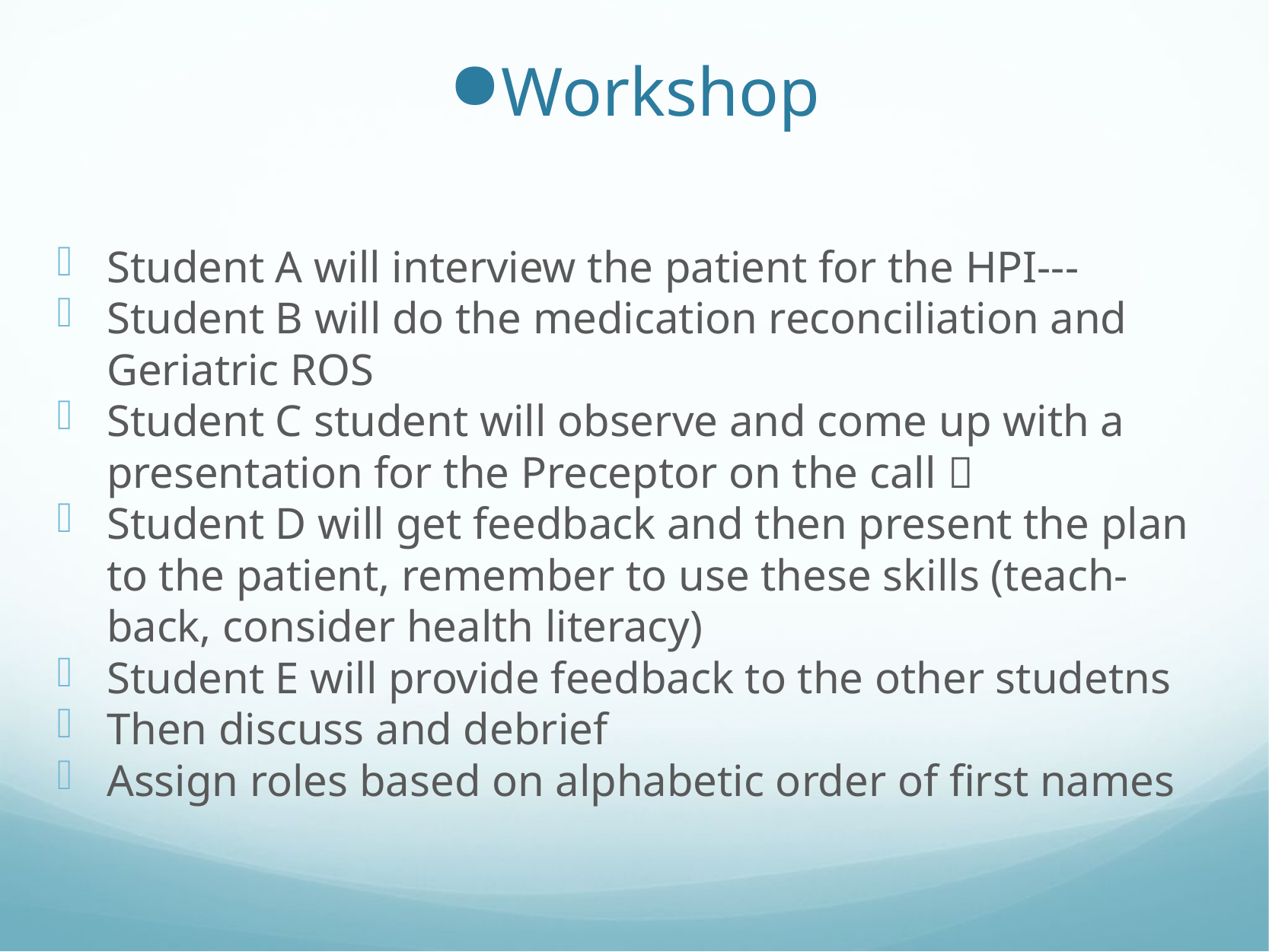

# Workshop
Student A will interview the patient for the HPI---
Student B will do the medication reconciliation and Geriatric ROS
Student C student will observe and come up with a presentation for the Preceptor on the call 
Student D will get feedback and then present the plan to the patient, remember to use these skills (teach-back, consider health literacy)
Student E will provide feedback to the other studetns
Then discuss and debrief
Assign roles based on alphabetic order of first names
