# Supplementary material for: Geriatric Telehealth: A Standardized Patient Case for Medical Students
Source: MedEdPORTAL. 2023 Sep 12;19:11345. doi: 10.15766/mep_2374-8265.11345 (PMC10495538; doi:10.15766/mep_2374-8265.11345)
Supplement: Supplementary file 1 — Pre- and Postsurvey.docxGeriatric Telehealth Didactic.pptxFacilitator and SP Guide.docxStudent Guide.docx [file mep_2374-8265.11345-s001.zip › C. Facilitator and SP Guide.docx]

**Geriatrics Telehealth Standardized Patient Activity—Provide this Entire Package to the SPs and Facilitators**

**Student Assignments**

Students will be in groups of 4-5. Each student should have an assignment:

1. **Student A** will be assigned to set the agenda with the patient, elicit questions that patient has, ensure appropriate lighting and hearing, triage the problems, and perform a history of the primary medical problem.
2. **Student B** will be assigned a geriatric review of systems and medication reconciliation.
3. **Student C** will perform a Presentation to the Preceptor including an Assessment and Plan. They will be asked to present in a problem-based format.
4. **Student D** will provide the information to the Patient in a patient friendly format. They will be encouraged to use Teach Back.
5. **Student E** will provide feedback to the other students.

If there are fewer students then roles can be combined and all the students will be asked to provide each other with feedback. For example, if there are two students, student A can cover roles A and B (10-20 minutes), and student B will cover roles of C and D (10-20 minutes). Then both students can provide each other with feedback. The facilitator and SP will provide student feedback regardless of the number of students.

**Structure of Session: Suggested Timeline**

Pretest: 5 minutes

Didactics on Geriatric Patient Care History Taking with Focus on Telehealth: 15-20 minutes

SP Session Introduction with Student Preparation of the Workshop (Provide the Student Document, Appendix D): 10 minutes

SP Interactions 5 min-10/student or role with patient: 30-45 minutes

Debrief with feedback SP, facilitator: 15 minutes

Posttest: 5 minutes

1.5-2 hour total

**Standardized Patient Information/Case Background**

You are Mr(s). Smith, an 80 year old who is scheduled via video-visit for 3 month follow up with your primary care physician. You are starting to experience some cognitive decline so you have recently given up driving. Your daughter manages your medication (fills pill box) and has been asking to help oversee your finances. The students should attempt to do teach-back with you at the end of the visit. You should be able to remember some but not all of what had been discussed (up to you what and how much!)

Q. Student should ensure that patient can hear and see them ok?

A. Yes, I can hear and see you (unless it’s not true! Then work with student to improve sound and lighting).

Q. Student should ask if anyone else needs to be on the call or is present?

A. My daughter wanted to be here but she’s at work today.

Q. Student should ask what patient hopes to discuss today (or another similar question to help set the agenda)?

A. My pain hasn’t been good, especially at night and it’s hard for me to fall asleep. I’m wondering if I can take Aleve PM as my daughter takes it and it really helps her sleep. Would it be ok for me to try?

Q. Med Student should ask about what patient currently does for pain.

A. I take Aleve and tumeric. The student should note that the doctor recommended Tylenol 1300 mg BID last visit. The patient does not remember this discussion.

**Student A: Setting the agenda: The student should prioritize**

1. Chronic pain and insomnia: Again the patient notes difficulty falling asleep due to longstanding right knee discomfort (knee has been an issue for “as long as I can remember”), taking Aleve for pain (but should only state this if specifically asked). No redness, warmth, or swelling.
2. Possible memory loss (student should specifically ask about IADLs and ADLs): Daughter helping more with meds, no longer driving, did get lost and gave up driving recently. Still managing finances but daughter has been asking to take this over. When asked about memory:

A: “I’m not like I used to be but that’s just part of getting older!”

1. Review of chronic medical problems

Q: How is your blood pressure?

A: I am not sure. I don’t check it.

Q: Have you had any weight loss?

A: I think I may have lost weight.

Q: Are you eating three meals?

A: I skip lunch, I just get busy and forget and am not hungry.

Q: Are you doing Ensure?

A: Oh I forgot about those.

Q: Any issues with bowels, constipation?

A: No miralax really works.

Q: Any chest pain, shortness of breath? Are you exercising?

A: No issues. I haven’t been exercising.

Q: Alcohol use

A: I don’t drink alcohol.

Q: Caffeine use

A: I drink one cup of coffee in the morning and iced tea with dinner.

**Student B: This student will ask some questions as part of a Review of Systems as well as perform medication reconciliation. Here are the answers to their questions:**

Q: Any issues with your bowels, such as constipation?

A: Bowels moving regularly, no constipation or diarrhea

Q: Any issues with your bladder, such as leakage or urgency?

A: No bladder leakage, incontinence, urgency but has nocturia 2-3x

Q: How is your sleep?

A: Not sleeping well due to discomfort. Does ‘doze’ sometimes after lunch.

Q: How is your memory?

A: Having more trouble remembering to pay bills, daughter wants to take over this task, has given up driving since last visit as got lost while trying to get home from a friend’s house and it was very upsetting. Daughter has started filling pill box.

Q: How is your mood?

A: Feeling a bit more anxious lately, worried about the virus. Not able to see family as regularly and more isolated but daughter very involved. Denies depression or need for any medication.

Q: Do you have any pain:

A: See responses in HPI.

Q: Have you lost or gained any weight?

A: Weight: Perhaps has lost a few pounds since last visit. No longer drinking Ensure as forgot. Also often skips lunch as forgets to eat.

Q: What are your medications? (Student should ask specifically about each medication.)

A: “My daughter left me the list. She fills the pill box!”

*Tylenol 1300 mg BID—not taking at all*

*Crestor 5 mg daily—taking once daily*

*Aspirin 81 mg daily—taking once daily*

*HCTZ 25 mg daily—taking once daily*

*Losartan 100 mg daily—taking once daily*

*Amlodipine 10 mg daily—taking once daily*

*Metoprolol XL 50 mg daily—taking* ***twice*** *a day (note this is incorrect)*

*Citracal+D petites 2 tabs twice a day—taking twice a day*

Miralax 17 gm daily titrated to daily bowel movements—taking daily

Q: Do you take any OTC herbs and supplements?

A: I take Aleve once daily in the morning and Tumeric daily.

Q: Do you use a pillbox?

A: Yes, my daughter fills my pill box once a week

**Student C. This student will present to the Facilitator.**

Student presentations may vary in style. The key is that they provide the necessary information in an organized fashion. Here is an example:

Mr(s). Smith, I am going to share with Dr. Wilson what we talked about today. Please let me know if I get anything wrong. Dr. Wilson, Mr(s). Smith and I addressed the following today.

I had a great talk with Mr(s.) Smith. Things are going ok, it’s harder right now with the virus as patient can’t see family and friends. Daughter is helping more which patient appreciates. We discussed the following issues--

1). Pain: We discussed the ongoing right knee pain from OA. Your note from last visit suggested Tylenol but Mr(s). Smith did not remember this discussion so s/he has been taking Aleve and tumeric in the morning. Daughter takes Aleve PM so patient wonders if s/he can take this at bedtime as it is hard for her/him to sleep due to the pain. S/he is not currently exercising. No warmth, redness, or swelling. I would recommend that we discontinue Aleve and start Tylenol, messaging daughter to place the Tylenol in the pillbox. I would also suggest Voltaren gel to be applied topically and physical therapy. Patient should avoid any PM medications going forward.

2) We reviewed patient’s activities and her/his daughter is helping more with her/his medications by filling a pill box. Mr(s). Smith has given up driving. Daughter would like to assist with finances too. Patient notes some memory changes, such as getting lost when driving. I would suggest that daughter assist with finances to ensure that there are no issues. I would suggest that we send the daughter a summary of today’s visit. Could consider a MOCA or MMSE to better evaluate.

3) We reviewed patient’s medications and noted that metoprolol is being taken twice a day (our list says once a day). I would suggest that take s/he metoprolol only once daily and that we ask daughter to check blood pressures.

4) Patient reports eating 2 meals a day, often skipping lunch and no longer doing Ensure. It is possible that s/he has lost weight but we do not have a recent measurement. I would suggest that patient’s daughter provides reminders for lunch and supplements.

Preceptor should provide guidance on student’s plan and/or approval.

**Student D: Student then should provide the following information to the patient.**

It was really great talking with you!

We have some ideas for your knee pain. We would like you to stop taking Aleve and use Tylenol instead. Tylenol is safer for older people. Please do not take any medications with PM as these are not safe for older people. We will prescribe a topical pain reliever for you to rub on your knee every 6 hours. Finally, would you be willing to have Physical Therapy? They can come to your house to work with you if you are interested. (Patient will be interested).

We are glad that you gave up driving and that your daughter is helping you. It sounds like she is willing to help you with your finances. We think this would be a good idea as sometimes patients who have trouble driving can have trouble with their bills. (Patient will be in agreement).

We would like you to take metoprolol only once a day. We will ask your daughter to make this change and check your blood pressure. We will send her a summary of what we discussed today. Would that be OK? (Patient will be very appreciative).

We will also ask her to weigh you to see if you have lost weight. It is important to eat 3 meals a day. We will ask your daughter to help with reminders. Also it may be good to restart the Ensure supplements that you were drinking as that seemed to help you maintain your weight.

For your difficulty with sleep we would suggest you avoid caffeine/tea in the evening and maybe even cut back in general or transition to decaf.

I know that’s a lot! Just to be sure we are on the same page, what will you do for your pain going forward?

What about for your metoprolol?

Specifically they should note

1. Concern for memory loss
2. Concern for improper dosing of metoprolol and reduction to once daily OR recommendation that BP and HR be checked
3. Concern for not taking acetaminophen as previously discussed and recommendation that patient take this at least at bedtime
4. Concern for Aleve (naproxen) and or Aleve (naproxen) PM and specifically recommend patient NOT take it until we try other measures

**Student E. Should provide feedback to Students A-D.** They should be encouraged to provide positive feedback of what the students did well with each component based on the introductory training session (PowerPoint).

**Debrief and Feedback**

**It is important to provide positive encouraging feedback! Start with asking the students to reflect on the experience:**

How did it go?

What went well?*

What challenges were encountered?*

How will the student change their approach based on this experience?

Is this a “common” geriatric encounter? Why or why not?

**Discuss the student responses and provide encouragement. Remind them that deliberate practice leads to improvement!**

*Feedback- Below are suggestions to guide feedback in the facilitator-led debrief for each student. Students should be encouraged to volunteer feedback and reflections on their own performance. When providing feedback to each other, students and standardized patients should emphasize positive behaviors along with any constructive advice.

Student A. Did the student:

- Ask the patient their concerns?
- Set the agenda?
- Ensure patient can hear and see?
- Redirect to questions if patient gets side-tracked?
- Review the patient’s medical issues and primary concern

Student B. Did the student:

- Cover the appropriate systems for a geriatric patient?
- Follow up with questions about weight loss (about meals, supplements)?
- Adapt to patient’s pace (if applicable)?
- Identify incorrect dosing of one of the metoprolol?
- Identify that the patient is taking turmeric?

Student C. Did the student note:

- Note concern for memory loss and recommendation of assessment
- Note concern for improper dosing of metoprolol and reduction to once daily OR recommendation that BP and HR be checked
- Note concern for not taking Apap and recommendation that patient take this at least at bedtime
- Note concern for Aleve PM and Aleve and specifically recommend patient NOT take it until we try other measures

Student D. Same as Student C but Student Should Ensure Patient Understanding:

- Did they use Teach Back?

Student E

- Did they provide specific feedback (both positive and constructive)?

**Case for Student (also to be provided to students)**

Mr(s). Smith is an 80-year-old who is scheduled via video-visit for 3 month follow up. You have your preceptor’s last clinic note as well as the patient’s medication list.

*Last clinic note:*

CC: Chronic pain, weight loss

Patient is a delightful patient presenting due to above problems.

1) Chronic pain due to right knee OA: Worse, knee aching constantly, using Tylenol sporadically

2) Weight loss: Stabilized, eating 3 meals a day with Ensure for snack

3) Osteoporosis: Had yearly Reclast, due for repeat in November

4) HTN: BP’s have been at goal

5) Constipation: Miralax effective

6) H/o CAD: Pt continues on aspirin, Crestor

Medications were reviewed and updated in EPIC.

Recent laboratory tests were reviewed.

SH: No alcohol, tobacco, drugs. Independent in IADLs, ADLs.

ROS: Negative except as per HPI

A/P

1) Chronic pain: Counseled on importance of scheduled Tylenol, start 1300 mg BID

2) Weight loss: Pt reports that she is not concerned with weight loss at present

3) Osteoporosis: Continue Reclast in November until 5 treatments

4) Constipation: Continue Miralax

5) HTN: Continue to monitor

6) H/o CAD: Continue aspirin, Crestor

Visit was 25 min, >50% spent on counseling/coordination of care-follow up in 3 months

Medication List

Tylenol 1300 mg BID

Crestor 5 mg daily

Aspirin 81 mg daily

HCTZ 25 mg daily

Losartan 100 mg daily

Amlodipine 10 mg daily

Metoprolol XL 50 mg daily

Citracal+D petites 2 tabs twice a day

Reclast once yearly

Miralax 17 gm daily titrated to daily bowel movements

Vitals for Today’s Visit: Not available
