# Supplementary material for: Geriatric Telehealth: A Standardized Patient Case for Medical Students
Source: MedEdPORTAL. 2023 Sep 12;19:11345. doi: 10.15766/mep_2374-8265.11345 (PMC10495538; doi:10.15766/mep_2374-8265.11345)
Supplement: Supplementary file 1 — Pre- and Postsurvey.docxGeriatric Telehealth Didactic.pptxFacilitator and SP Guide.docxStudent Guide.docx [file mep_2374-8265.11345-s001.zip › D. Student Guide.docx]

**Case for Student (also to be provided to students)**

Mr(s). Smith is an 80-year-old who is scheduled via video-visit for 3 month follow up. You have your preceptor’s last clinic note as well as the patient’s medication list.

*Last clinic note:*

CC: Chronic pain, weight loss

Patient is a delightful patient presenting due to above problems.

1) Chronic pain due to right knee OA: Worse, knee aching constantly, using Tylenol sporadically

2) Weight loss: Stabilized, eating 3 meals a day with Ensure for snack

3) Osteoporosis: Had yearly reclast, due for repeat in November

4) HTN: BP’s have been at goal

5) Constipation: Miralax effective

6) H/o CAD: Pt continues on aspirin, crestor

Medications were reviewed and updated in EPIC.

Recent laboratory tests were reviewed.

SH: No alcohol, tobacco, drugs. Independent in IADLs, ADLs.

ROS: Negative except as per HPI

A/P

1) Chronic pain: Counseled on importance of scheduled Tylenol, start 1300 mg BID

2) Weight loss: Pt reports that she is not concerned with weight loss at present

3) Osteoporosis: Continue reclast in November until 5 treatments

4) Constipation: Continue miralax

5) HTN: Continue to monitor

6) H/o CAD: Continue aspirin, crestor

Visit was 25 min, >50% spent on counseling/coordination of care-follow up in 3 months

Medication List

Tylenol 1300 mg BID

Crestor 5 mg daily

Aspirin 81 mg daily

HCTZ 25 mg daily

Losartan 100 mg daily

Amlodipine 10 mg daily

Metoprolol XL 50 mg daily

Citracal+D petites 2 tabs twice a day

Reclast once yearly

Miralax 17 gm daily titrated to daily bowel movements

Vitals for Today’s Visit: Not available
